# Systematic identification of genes involved in metabolic acid stress resistance in yeast and their potential as cancer targets

**DOI:** 10.1242/dmm.023374

**Published:** 2016-09-01

**Authors:** John J. Shin, Qurratulain Aftab, Pamela Austin, Jennifer A. McQueen, Tak Poon, Shu Chen Li, Barry P. Young, Calvin D. Roskelley, Christopher J. R. Loewen

**Affiliations:** Department of Cellular and Physiological Sciences, Life Sciences Institute, University of British Columbia, Vancouver, British Columbia, Canada V6T 1Z3

**Keywords:** AP-3 complex, Hap1 cells, Mitochondria, PAN complex, Intracellular acid stress, Metabolism

## Abstract

A hallmark of all primary and metastatic tumours is their high rate of glucose uptake and glycolysis. A consequence of the glycolytic phenotype is the accumulation of metabolic acid; hence, tumour cells experience considerable intracellular acid stress. To compensate, tumour cells upregulate acid pumps, which expel the metabolic acid into the surrounding tumour environment, resulting in alkalization of intracellular pH and acidification of the tumour microenvironment. Nevertheless, we have only a limited understanding of the consequences of altered intracellular pH on cell physiology, or of the genes and pathways that respond to metabolic acid stress. We have used yeast as a genetic model for metabolic acid stress with the rationale that the metabolic changes that occur in cancer that lead to intracellular acid stress are likely fundamental. Using a quantitative systems biology approach we identified 129 genes required for optimal growth under conditions of metabolic acid stress. We identified six highly conserved protein complexes with functions related to oxidative phosphorylation (mitochondrial respiratory chain complex III and IV), mitochondrial tRNA biosynthesis [glutamyl-tRNA(Gln) amidotransferase complex], histone methylation (Set1C–COMPASS), lysosome biogenesis (AP-3 adapter complex), and mRNA processing and P-body formation (PAN complex). We tested roles for two of these, AP-3 adapter complex and PAN deadenylase complex, in resistance to acid stress using a myeloid leukaemia-derived human cell line that we determined to be acid stress resistant. Loss of either complex inhibited growth of Hap1 cells at neutral pH and caused sensitivity to acid stress, indicating that AP-3 and PAN complexes are promising new targets in the treatment of cancer. Additionally, our data suggests that tumours may be genetically sensitized to acid stress and hence susceptible to acid stress-directed therapies, as many tumours accumulate mutations in mitochondrial respiratory chain complexes required for their proliferation.

## Abstract (precis)

**Summary:** Altered metabolism in tumours creates metabolic acid stress in tumour cells, which is a target for chemotherapeutics. We identify six new complexes with roles in resistance to metabolic acid stress.

## INTRODUCTION

Oncogenic transformation initiates dramatic changes in the primary metabolism of cancer cells that help enable their rapid proliferation and increased invasive capability, which ultimately leads to primary tumour formation and contributes to the formation of distant tumour lesions during metastasis. Almost universally associated with all primary and metastatic tumours is high glucose uptake and the glycolytic phenotype, which is associated with incredibly high rates of glycolysis (Gatenby and Gillies, 2004). These metabolic changes are a consequence of the poor oxygen conditions associated with the tumour microenvironment and are mediated initially by the HIF1 transcriptional response (Gatenby et al., 2007; Pouyssegur et al., 2006). However, through the process of somatic evolution, tumours undergo permanent metabolic changes that result in the persistence of high rates of glycolysis, even in the presence of oxygen, that is no longer HIF1-dependent (Gatenby and Gillies, 2004). This ‘aerobic’ glycolysis allows metastatic tumour cells to rapidly proliferate and move throughout the body.

A major cellular consequence of high levels of glycolysis is the accumulation of metabolic acid, primarily in the form of lactic acid and hydrogen ions; therefore, cancer cells experience considerable intracellular acid stress. In response to decreased intracellular pH (pH_i_), acid pumps in the plasma membrane are upregulated to expel metabolic acid out of the cell. The two major classes of these pumps are the sodium-hydrogen exchangers (NHEs) and the monocarboxylate transporters (MCTs). Tumour cells also upregulate a relay system involving the carbonic anhydrase enzyme and bicarbonate transporter to reduce accumulation of intracellular carbon dioxide and also the resulting acid (Pouyssegur et al., 2006). These adaptations, combined, are thought to keep the pH_i_ of tumour cells from becoming acidic after the oncogenic changeover to the glycolytic phenotype (Gillies et al., 1990).

The finding that tumour cells undergo intracellular acid stress and upregulate pumps to compensate implies that these are potential therapeutic targets in the treatment of cancer (Cardone et al., 2005; Gatenby et al., 2007; Izumi et al., 2003; Lagana et al., 2000; Reshkin et al., 2000). Indeed, upregulation of NHE1 is observed in tumour cells (McLean et al., 2000; Ober and Pardee, 1987) and overexpression of NHE1 in fibroblasts induces the glycolytic phenotype and drives malignant transformation (Reshkin et al., 2000), suggesting that pH changes in tumour cells are a primary factor in the progression of cancer. Additionally, downregulation of NHE1 in tumour cells has profound inhibitory effects on motility, invasiveness and tumourigenicity (Kumar et al., 2009; Li et al., 2009; Yang et al., 2010). Pharmacologic NHE1 inhibitors have been tested in phase II and III clinical trials for the treatment of heart failure (Karmazyn, 2001), and they are potentially promising drugs for the treatment of cancer (Cardone et al., 2005). Another proton pump, the vacuolar (V)-ATPase, is also upregulated and redirected to the plasma membrane in highly metastatic tumour cells, where it is required for the cells' highly invasive and migratory behaviour (Martinez-Zaguilan et al., 1993; Sennoune et al., 2004), making V-ATPase another potential therapeutic target, with drugs currently being developed (Fais et al., 2007; Perez-Sayans et al., 2009).

Our previous work studying factors that regulate phospholipid metabolism in budding yeast uncovered that pH_i_ is a discrete signal regulating expression of phospholipid synthesis genes, enabling coupling of nutrient availability to membrane biogenesis and cell growth (Young et al., 2010). Yeast have a remarkable capacity to maintain neutral pH_i_ even under conditions of extreme extracellular acid stress, because they express a fungal-specific P2-type plasma membrane H^+^-ATPase, Pma1, which pumps protons generated by metabolism out of the cell (Ferreira et al., 2001). Pma1 accounts for nearly one third of total plasma membrane protein and we discovered that a yeast mutant with decreased expression (called *pma1-007*) was unable to maintain normal physiological pH_i_ under conditions of extracellular acid stress, resulting in a predictable decrease in pH_i_ upon extracellular acidification (Young et al., 2010). Therefore, using the *pma1-007* mutant it was now possible to introduce intracellular acid stress systematically, simply by decreasing the pH of the growth medium, and hence, to screen for previously uncharacterised acid stress resistance genes. Here, we report the identification of 129 acid stress resistance genes in yeast, identify six highly conserved acid stress resistance complexes, and confirm that two complexes, AP-3 and PAN are involved in acid stress resistance in a human myeloid leukaemia-derived cell line that we have developed as a model for acid stress resistance in cancer.

## RESULTS

### A systematic genetic screen for intracellular acid stress resistance genes

**Fig. 1.**
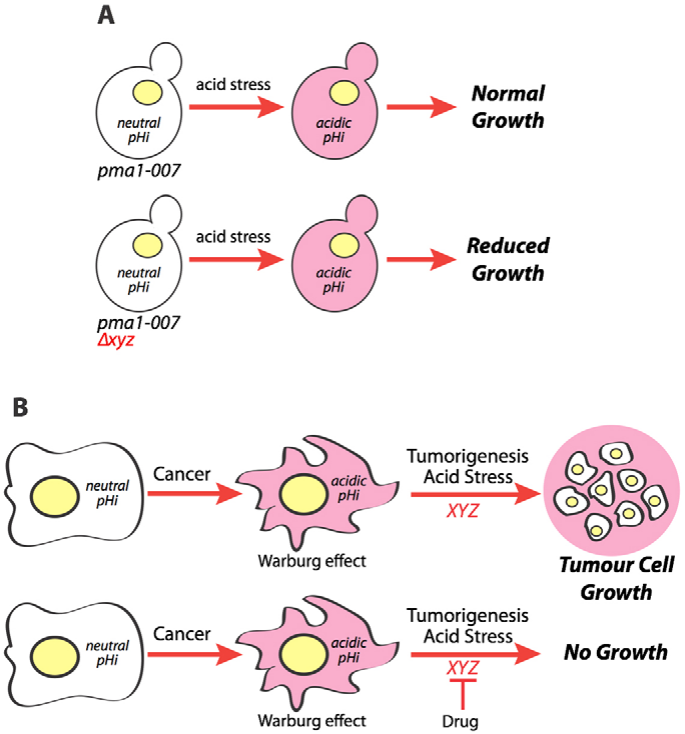
**Yeast as a model to newly identify acid stress resistance genes in cancer.** (A) Genes (*XYZ*) required for survival of acid stress when deleted in the acid stress-sensitized yeast strain *pma1-007* should cause reduced growth in conditions of acid stress. (B) These same genes might be involved in tumour cell survival of acid stress in the tumour environment and represent potential therapeutic targets in the treatment of cancer.

We devised a screening strategy to identify genes that were required for cell survival of acid stress (Fig. 1). In this strategy, growth of the *pma1-007* mutant on acidified medium results in lowering of pH_i_, causing intracellular acid stress, which mimics at least one aspect of the metabolic transformation to the glycolytic phenotype that occurs in cancer. Deletion of genes that are required for survival of acid stress should reduce growth of the *pma1-007* mutant under acid-stressing conditions. Hence, such genes might represent potential acid stress therapeutic targets in cancer.

**Fig. 2.**
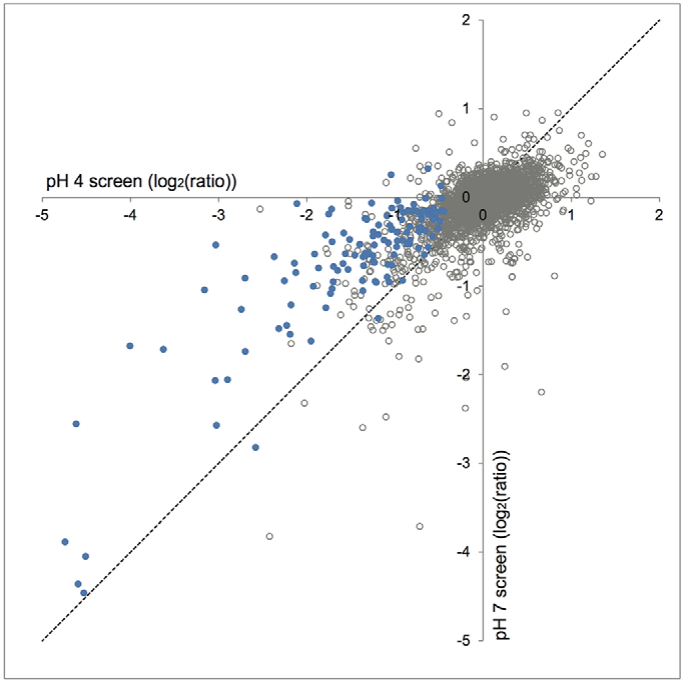
**Identification of 129 genes required for resistance to acid stress in yeast.** Plotted is the log_2_ of the ratio of growth of double mutants with *pma1-007* relative to their single mutant controls for genetic screens performed at pH 4 and 7. Blue dots indicate double mutants identified to have decreased growth relative to single mutant controls in the pH 4 screen (*P*<0.05 by Student's *t*-test, *n*=3). Grey rings indicate double mutants with non-significant changes in growth in the pH 4 screen.

Using synthetic genetic array (SGA) technology, we constructed double-mutant haploid yeast carrying the *pma1-007* hypomorphic allele of the P-type H^+^-ATPase of the plasma membrane and null alleles of ∼4800 other nonessential genes. We then quantified growth of the double mutants relative to single mutant controls on acidic medium, pH 3, 4 and 5, and neutral pH 7 medium, using Balony software (Table S1) (Young and Loewen, 2013). For the systematic identification of acid stress resistance genes we analysed the screen performed at pH 4 because this screen showed better, more uniform growth of the yeast high-density arrays than at pH 3 (data not shown), and at pH 4, the *pma1-007* mutant showed substantially reduced pH_i_ compared with pH 5 (∼6.9 at pH 4 vs ∼7.05 at pH 5, compared with ∼7.1 for wild type; Young et al., 2010). We identified 129 double mutants that showed reduced growth at pH 4 relative to the single-mutant controls according to statistical thresholds set using Balony software (Table S2). We plotted the ratio of growth of double mutants versus single mutants for screens at both pH 4 and 7 (Fig. 2). The bulk of the double mutants were not affected by acid stress as they grew similarly to the single mutant controls and hence, their ratios clustered near zero on the graph (grey rings). Interestingly, nearly all of the 129 double mutants that showed slow growth phenotypes at pH 4 (blue dots) landed above the diagonal on the graph, indicating that their growth was improved at pH 7. Thus, these 129 genes were likely involved in resistance to acid stress.

**Fig. 3.**
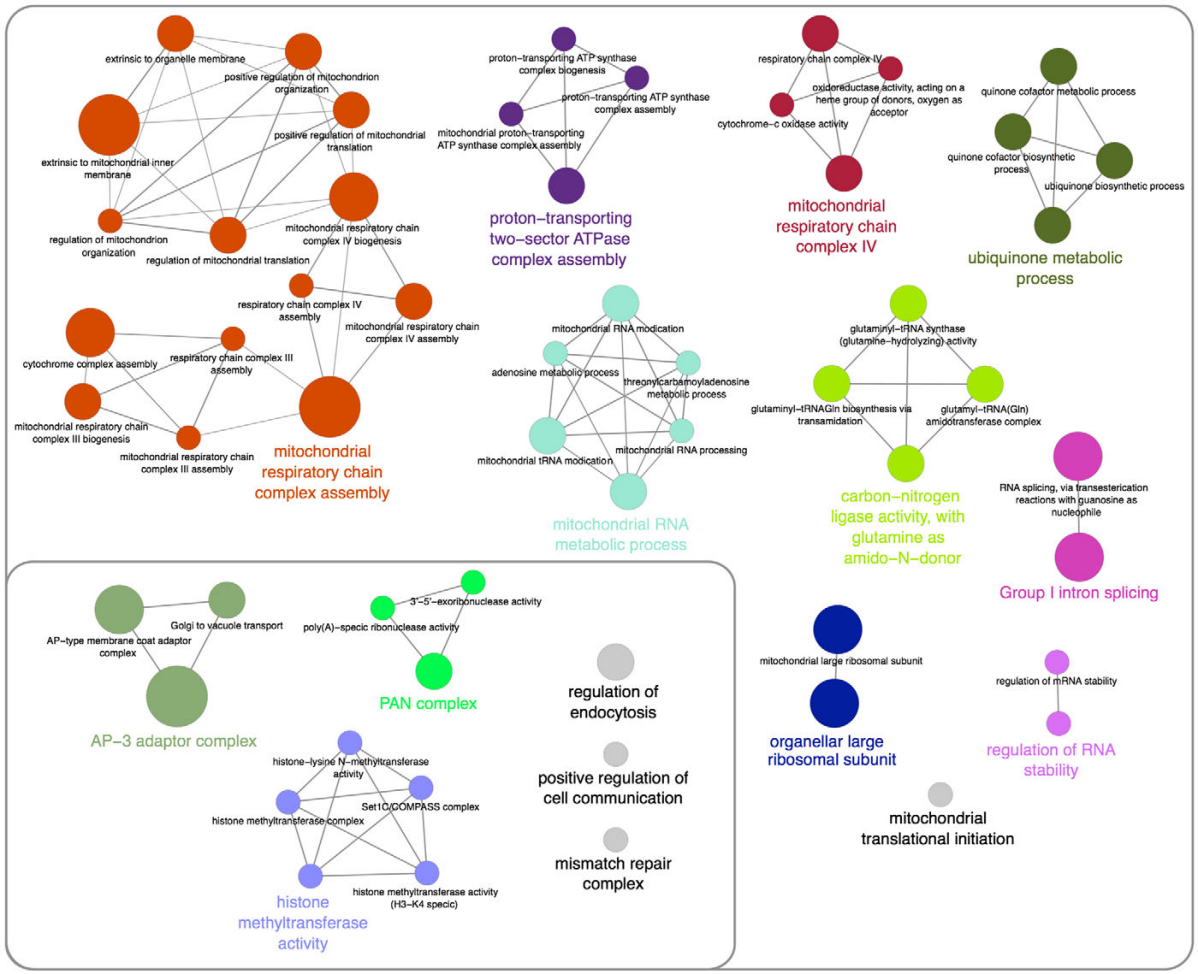
**Functional enrichment in the yeast acid stress screen at pH 4.** Enrichment for gene ontology (GO) groups was done using the ClueGO plugin and Cytoscape software. Nodes are coloured according to grouping of related functions by statistically significant association of related GO terms (ungrouped terms depicted as grey nodes) and groups are labelled according to the most significant term of the group. Node size corresponds to the significance of each GO term in the network and edges indicate statistically significant associations between GO terms. Functions related to mitochondria are shown in the larger area whereas other non-mitochondrial functions are shown in the smaller outlined area.

To reveal functional enrichment within this acid stress resistance gene set we queried the Gene Ontology annotations database using the ClueGO plugin for Cytoscape (Bindea et al., 2009; Shannon et al., 2003). Multiple functions related to mitochondria, including respiration, ATP synthesis, RNA processing/metabolism and translation were enriched for (Fig. 3; Fig. S1, Table S3). Other non-mitochondrial processes were also enriched for, including AP-3 adapter complex-mediated Golgi to vacuole transport, poly(A)-specific PAN complex-mediated RNA processing, and histone methylation via the Set1C–COMPASS complex. Importantly, these functions are highly conserved between yeast and humans, indicating that the mechanisms governing resistance to intracellular acid stress are conserved.

### Acid stress resistance complex identification

**Fig. 4.**
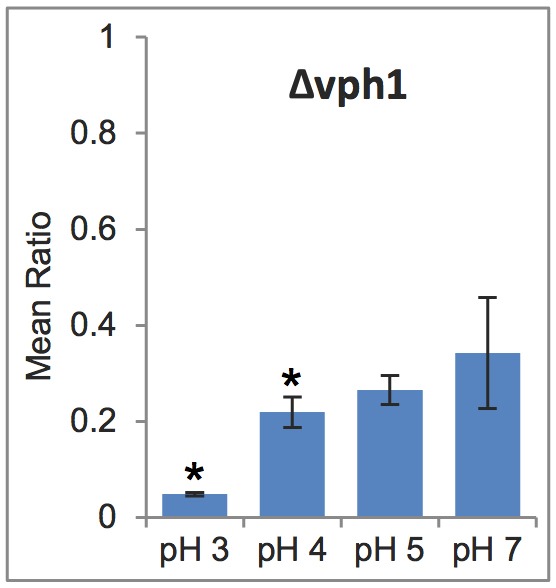
**The vacuolar ATPase subunit Vph1 is an acid stress resistance gene.** The mean ratio of growth of the Δ*vph1 pma1-007* double mutant relative to the Δ*vph1* single mutant is plotted for screens performed at the indicated pH values (*n*=3). Note that at pH 3 the double mutant is extremely slow growing and at pH 7 it is not rescued fully to the growth of the Δ*vph1* single mutant (i.e. mean ratio<1). This is likely because loss of vacuolar ATPase function also results in intracellular acid stress (Young et al., 2010). Error bars indicate s.e.m. *P<0.05 vs pH 7 by Student's *t*-test.

Given the known role for the V-ATPase complex in acid stress resistance in cancer (Martinez-Zaguilan et al., 1993; Sennoune et al., 2004), we looked for yeast V-ATPase components in our dataset. There are 13 V-ATPase mutants present in our yeast deletion collection array. Of these, loss of *VPH1* encoding the ‘a’ subunit of the V_0_ domain of the ATPase greatly sensitized yeast to acid stress and was identified in 3/3 replicates (Fig. 4). Loss of Vph1 abolishes V-ATPase activity and proton pumping, preventing vacuolar acidification (Manolson et al., 1992). We also identified *VMA1*, *VMA6* and *VMA16* in 2/2 replicates, indicating that in each of these screens both the control and experimental spots were absent in one out of the three biological replicates, but in the other two replicates loss of these genes sensitized the yeast to acid stress. Additionally, we identified *VMA4* in 1/3 replicates and *VMA5* in 1/2 replicates; in both cases the *VMA4* and *VMA5* single mutant controls were so slow-growing that they were below the limit of our ability to reliably detect genetic interactions in more than one replicate. For the remainder of the V-ATPase genes in our array (*VMA2*, *VMA3*, *VMA8*, *VMA10*, *VMA11*, *VMA13*), the single mutant control strains were not alive in the final selection stage and could not be analysed (with the exception of the *VMA7* gene, which was linked to the *PMA1* locus and could not be analysed). V-ATPase mutants are known to have greatly reduced fitness, so it is not surprising that many of these mutants did not make it through the SGA protocol. Therefore, with a considerable degree of confidence we feel we can extend the identification of V-ATPase mutants to four genes (*VPH1*, *VMA1*, *VMA6*, *VMA16*), and with a fair degree of confidence to a total of six genes (adding *VMA4*, *VMA5*). Thus, we are confident that disrupting V-ATPase function makes yeast more susceptible to intracellular acid stress. This is consistent with known roles for the V-ATPases in collaborating with Pma1 in pH regulation (Martínez-Muñoz and Kane, 2008). Hence, identification of the V-ATPase also suggested that our screening approach might be relevant to acid stress resistance in cancer.

**Fig. 5.**
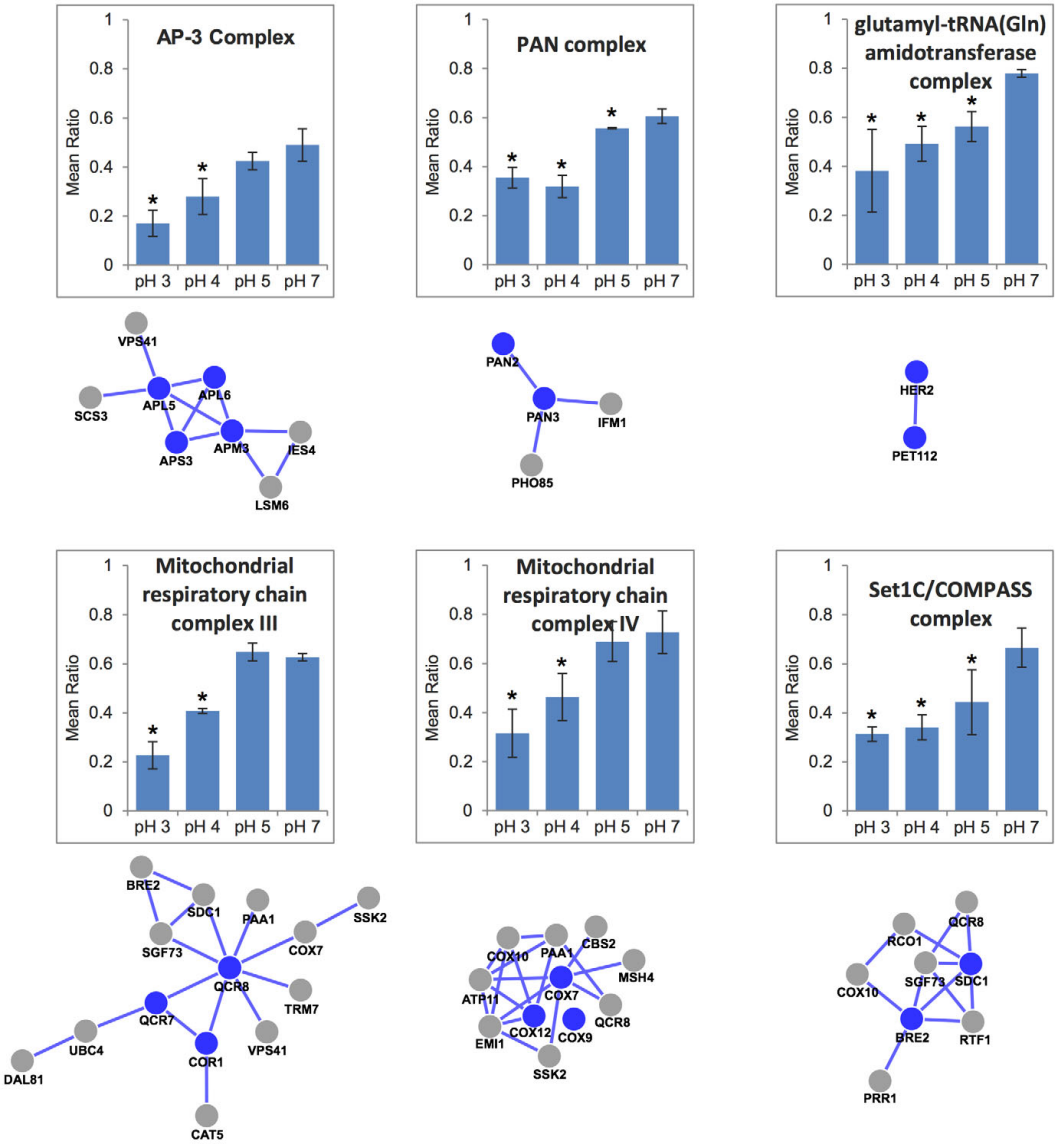
**Identification of acid stress resistance complexes.** Complexes were identified using the GeneMania plugin for Cytoscape using the pH 4 acid stress screen gene set. Blue nodes indicate proteins identified in the screen that are subunits of the complexes, whereas grey nodes indicate proteins identified in the screen that interact with the complexes. Edges indicate protein–protein interactions. Graphs show the average for the complexes of the mean ratios of growth for double mutants relative to single mutant controls (as in Fig. 4) for the screens performed at the indicated pH (*n*=3 for each subunit). Error bars indicate s.e.m. *P<0.05 vs pH 7 by Student's *t*-test.

We next searched our pH 4 screen dataset for individual protein complexes as these represented logical potential therapeutic targets of acid stress in cancer. We identified 16 acid stress resistance genes belonging to six individual annotated protein complexes (Fig. 5, blue nodes). These complexes were highly conserved from yeast to humans and were crucial to many of the processes identified by gene ontology analysis (Fig. 3). Furthermore, four of the six acid stress resistance complexes identified in the pH 4 screen (AP-3, PAN, Complex III and Complex IV) were also identified in both the pH 5 and pH 7 screens, whereas the remaining two [glutamyl-tRNA(Gln) amidotransferase complex and Set1C–COMPASS complex] were additionally identified only in the pH 5 screen (Table S1). Using our screen data from each pH condition, we plotted the average of the mean ratios of growth for the genes corresponding to each of the complexes under conditions of increasing acid stress (Fig. 5). For all six complexes, their disruption led to substantially decreased growth under acid stress, supporting that complex function in each case was linked to cellular resistance to acid stress. By examining the protein–protein interaction network within our pH 4 screen dataset (Fig. S2) we also uncovered additional proteins that interacted with each of the six complexes, further expanding the number of potential acid stress therapeutic targets related to these complexes from 16 to 31 genes (Fig. 5, grey nodes). We selected AP-3 and PAN complexes for further validation using yeast spot assays and found that loss of any of the subunits of either the AP-3 or PAN complex sensitized yeast to growth under acid stress, consistent with our array-based growth results (Fig. S3).

### Roles for the AP-3 adapter and PAN deadenylase complexes in resistance to acid stress in human cancer cells

We were now interested in assessing roles for these complexes in resistance to acid stress in human cells, particularly those that could potentially be therapeutically useful targets. We chose to use the Hap1 human haploid fibroblastoid cell line, derived from a chronic myelogenous leukemic line KMB-7 isolated from a 39-year-old man with chronic myeloid leukaemia in blast crisis (Essletzbichler et al., 2014; Kotecki et al., 1999). Hap1 cells harbour the BCR-ABL oncogenic fusion and have been engineered to be haploid in nature (whereas KMB-7 is near-haploid). We chose the Hap1 cell line because they are malignant neoplastic cells and because its haploidy makes it highly amenable to genetic studies, as recessive mutations are not masked by a second copy of each gene, allowing for increased phenotypic penetrance. It is also likely a good choice for studying the effects of intracellular acid stress. This is because significant acidification of the bone marrow micro-environment (as low as pH ∼6.5) occurs during intensive blast production in the progression of leukaemias (Mortensen et al., 1998), suggesting that intracellular acid stress resistance mechanisms are likely required for progression of blood-born cancers and are thus likely also in place in Hap1 cells (see below).

**Fig. 6.**
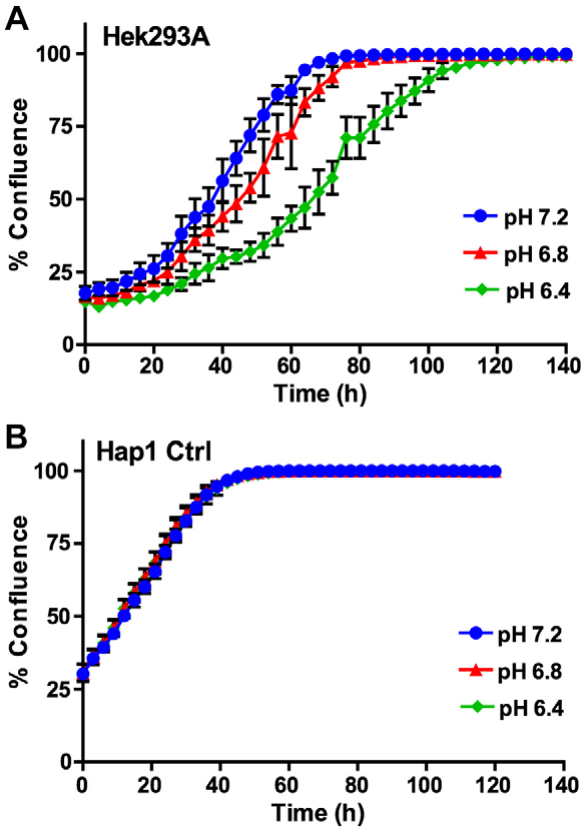
**Growth of HEK293 cells is sensitive to acid stress whereas Hap1 cell growth is not over the pH range 6.4-7.2.** HEK293A cells (A) and Hap1 control cells (B) were plated in 96-well plates at a starting density of 10,000 cells/well. After allowing cells to adhere to the plate for 2 h, cells were put into growth media buffered at pH 7.2, 6.8 or 6.4. Cells were then monitored for confluence in an IncuCyte imager over the timescale shown. Results from six replicate wells are shown. Error bars indicate standard deviation.

To begin we wanted to test conditions for introducing acid stress in cell culture and to characterize the response of the Hap1 cell line relative to a non-cancerous human cell line. We chose HEK293 cells for the comparison because they are a robust adherent human cell line that grows well in culture. We adjusted media pH within the range of 6.4 to 7.2, because acidification over this range has been shown to substantially decrease pH_i_ in adherent cells in culture (Balgi et al., 2011). Using an IncuCyte cell imager we monitored growth of HEK293 cells over time in media buffered at pH 6.4, 6.8 and 7.2. Relative to pH 7.2 media, cell growth in pH 6.8 and 6.4 media was successively slowed over the time course of almost six days (Fig. 6A). We now compared this to growth of Hap1 cells under these same conditions. In contrast to HEK293 cells, Hap1 cells showed no decrease in growth rate in acidified medium over the time course (Fig. 6B). Hap1 growth rate was also considerably faster than the HEK293 cells, reaching 95% confluence at ∼39 h versus 64 h for HEK293 in pH 7.2 medium. We measured viability of the HEK293 cells under acid stress by PI exclusion assay and found no decrease in viability, indicating the decrease in cell growth was a result of a slowed cell cycle (Fig. S5). Thus, over the media pH range studied, Hap1 cell growth seemed to be resistant to acid stress, confirming that it was a good model cancer cell line to study mechanisms of acid stress resistance.

We now focused on two of the acid stress resistance complexes; the AP-3 and PAN complexes. Loss of function of AP-3 is the cause of Hermansky–Pudlak syndrome, which results in a mild bleeding disorder (Dell'Angelica, 2009), suggesting AP-3 could be targeted as a therapeutic. PAN complex is also likely a reasonable therapeutic target given its function is partially redundant with another poly(A) deadenylase complex, Ccr4–Not1 (see Discussion for details) (Yamashita et al., 2005). We analysed deletions of the delta subunit of the AP-3 complex (AP3D1) or the Pan2 subunit of the PAN complex in two Hap1 cell lines made using the CRISPR–Cas9 method. Disruption of the AP3D1 gene results in loss of AP-3 complex function and is the causative genetic lesion behind the *mocha* mouse (Kantheti et al., 1998). Pan2 is the catalytic subunit of the PAN complex and hence is required for PAN deadenylase activity (Schäfer et al., 2014). Gene disruption in each cell line was confirmed by western blot analysis (Fig. S4).

**Fig. 7.**
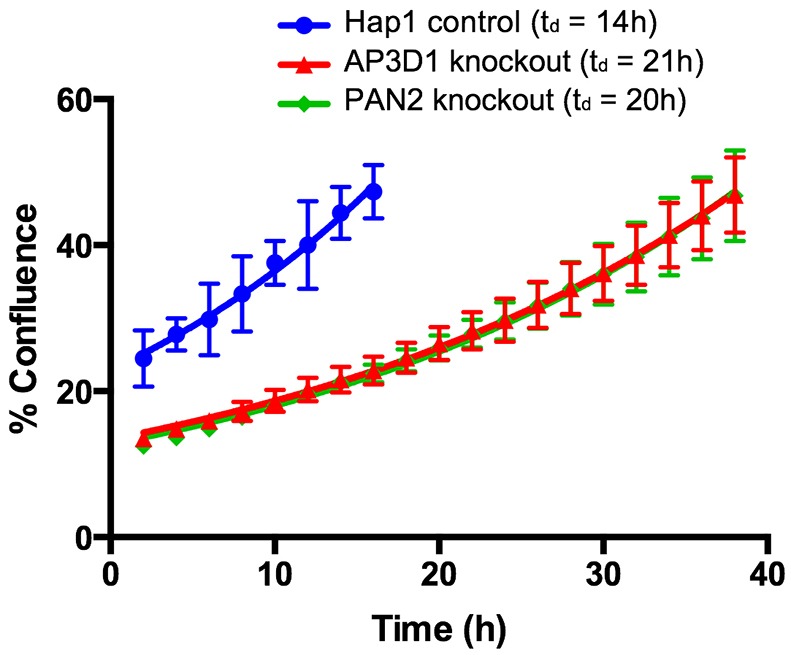
**Knockout of AP-3 and PAN complexes reduces the growth rate of Hap1 cells.** Plotted is the percentage confluence for the given cell lines grown in buffered pH 7.2 media for the given times, determined using an IncuCyte cell imager. Curves were fit using non-linear regression with a single exponent and doubling times (t_d_) are shown on the graph. Doubling times for AP3D1 and PAN2 knockout cells are significantly different than Hap1 control cells (*P*<0.0001 by Student's *t*-test, *n*=8), but are not significantly different from each other. Error bars indicate s.e.m.

First, to determine if disruption of AP-3 and PAN complexes affected cell growth rate, we performed growth assays using media buffered at pH 7.2. Growth was assessed in real-time over 72 h using an IncuCyte cell imager (Fig. S6). To determine growth rates from the growth curves we performed nonlinear regression analysis using data from the initial time points up until the cells reached ∼50% confluence (Fig. 7), as this region of the curves seemed to show an exponential increase in confluence. From these curves we determined doubling times for control, AP3D1 and PAN2 knockout cells of 15 h, 21 h and 20 h, respectively (*P*<0.0001 between knockouts and control; the difference between AP3D1 and PAN2 knockouts was not significant). Thus, loss of AP-3 and PAN complex function negatively impacted the rate of Hap1 cell growth in culture, suggesting that these complexes were promising cancer targets.

**Fig. 8.**
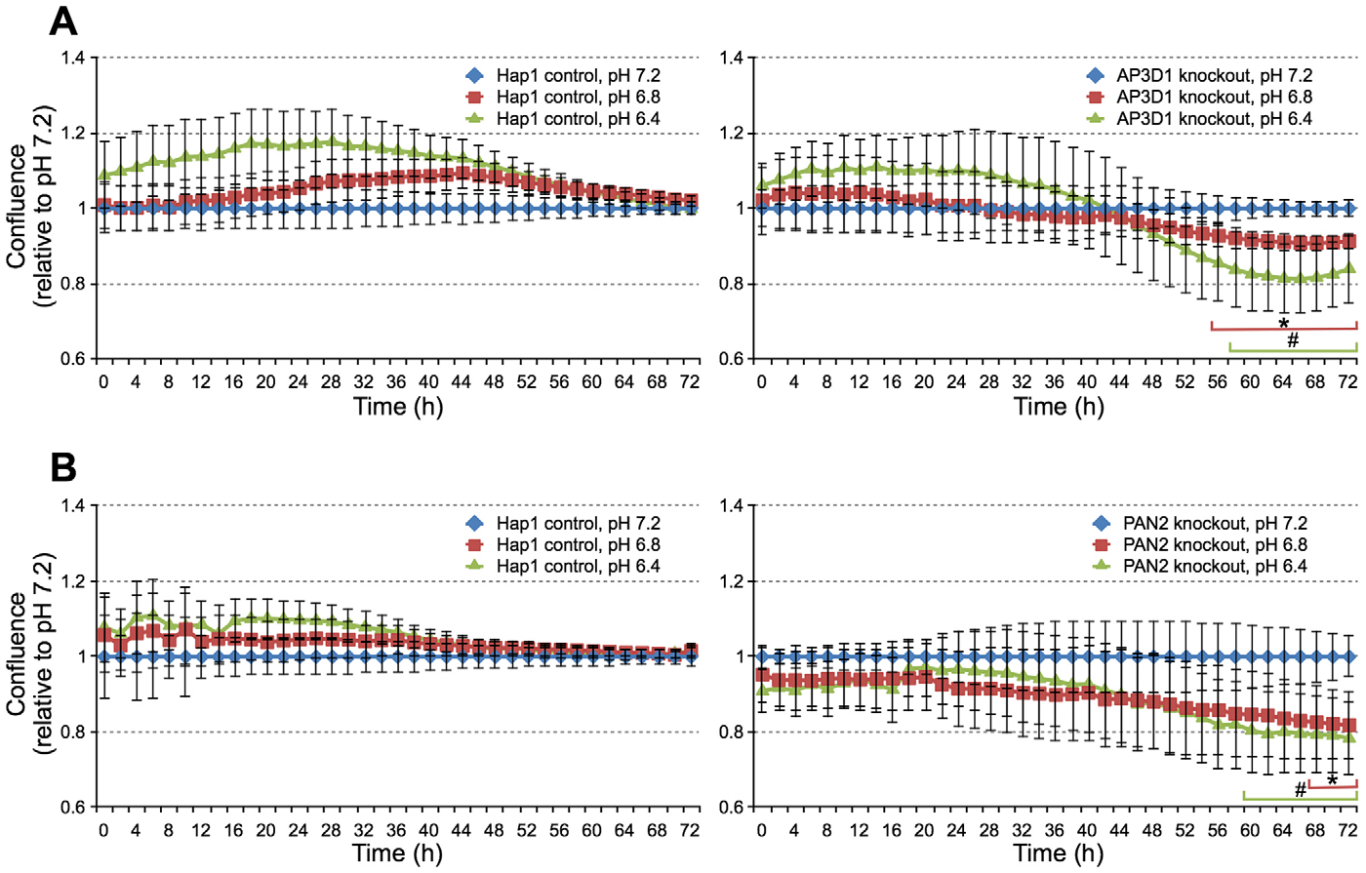
**AP-3 and PAN complex knockouts show sensitivity to acid stress.** (A) Growth of AP3D1 knockout cells versus Hap1 control cells at the given pH values. (B) Growth of PAN2 knockout cells versus Hap1 control cells at the given pH values. In each graph in A and B, growth measured using an IncuCyte imager at each pH is normalized to growth at pH 7.2 [Confluence (relative to pH 7.2)]. Statistically significant decreases in growth relative to pH 7.2 are marked on the graphs (*P*<0.05, * pH 6.8 vs 7.2; # pH 6.4 vs 7.2 by Student's *t*-test; *n*=6-8). Error bars indicate s.e.m.

We next investigated a role for the AP-3 and PAN complexes in resistance to acid stress in culture. Again we monitored growth of cells in media buffered at pH 7.2, 6.8 and 6.4 over 72 h using an IncuCyte imager. Because of the difference in growth rate of AP3D1 and PAN2 knockout cells relative to control Hap1 cells, we normalized growth of each cell line at each pH to growth at pH 7.2 in order to determine the effects of acid stress (Fig. 8). For Hap1 control cells, we observed no significant decrease in growth under acid stressing conditions at pH 6.8 or 6.4 relative to pH 7.2 over the 72 h time course of the experiment, as we had observed before. In contrast, in both AP3D1 and PAN2 knockout cells we observed a statistically significant decrease within the third day of growth under conditions of acid stress, pH 6.8 and 6.4. On the third day of growth these cells were >50% confluent, compared with Hap1 control cells, which reached 50% confluence at ∼15 h, within the first day (Fig. 7). However, the Hap1 control cells showed no growth defects under conditions of acid stress at this or higher cell density. Interestingly, the acid stress sensitivity of the AP-3 and PAN knockout cells was different than the effects of acid stress on HEK293 cells, which resulted in decreased exponential phase growth (see Discussion).

We next investigated whether the decrease in growth rate, and particularly the acid stress sensitivity, of the AP-3 and PAN complex knockout cells resulted from increased apoptosis, as has been found for HL-60 human promyelocytic leukaemia cells under conditions of acid stress (Park et al., 1996), which occurs via Bax-mediated caspase activation and PARP cleavage (Park et al., 1999). We measured cell proliferation by MTT assay at physiological pH and found that similarly to the cell confluence data generated using the IncuCyte imager, the MTT assay also showed decreased proliferation of both the AP-3 and PAN knockout cells (Fig. S7), supporting that loss of either of these complexes generally slowed down Hap1 growth rate. To assess apoptosis, we measured PI exclusion using a Cellomics Array Scan in the mutants under acid stressing conditions. We did not find any indication of acid stress-induced apoptosis occurring in either of the mutants or the control at pH 6.4, 6.8 and 7.2 up to 72 h in culture (Fig. S8), indicating that apoptosis did not contribute to the decreased rates of proliferation in the mutants. We also did not detect any changes in cell morphology in the mutants or under acid stressing conditions (Fig. S9), further supporting the viability of the cells. Thus, loss of AP-3 and PAN complex function reduced the rate of cell growth under normal and acid stressing conditions.

## DISCUSSION

Using a systematic whole-genome screening approach in budding yeast we identified 129 genes that were involved in resistance to intracellular acid stress. We uncovered that essential highly conserved mitochondrial processes required for cellular respiration were enriched for, as well as lysosome biogenesis, mRNA processing and chromatin. We identified six protein complexes in our dataset linked to 31 genes in total that we propose represent potential therapeutic acid stress targets in cancer. We investigated roles for two of them, the AP-3 adapter complex and the PAN deadenylation complex, in resistance to acid stress in human cells. Loss of either of these complexes resulted in decreased growth rate of Hap1 myeloid leukaemia cells in culture and sensitivity to acid stress, indicating that these were potentially novel targets in the treatment of cancer. Although the mechanisms by which AP-3 and PAN complexes function in acid stress resistance are unknown, that these functions appear to be conserved in both yeast and humans suggests that cellular acid stress resistance mechanisms are likely present in all eukaryotes and are a fundamental aspect of cell physiology.

### AP-3 complex as a target of intracellular acid stress resistance in cancer

The AP-3 adapter complex is a heterotetramer of four non-homologous subunits that are conserved from yeast to humans and which plays roles in intracellular membrane traffic and biogenesis of lysosomes and lysosome-related organelles (Dell'Angelica, 2009). We identified all four subunits in our screen (Fig. 5). AP-3 is mutated in Hermansky–Pudlak syndrome, a rare recessive disorder that results in albinism and mild bleeding disorders (Dell'Angelica, 2009). The AP-3 complex is required for lysosomal transport of lysosomal membrane proteins (LIMPs and LAMPs) (Le Borgne et al., 1998); lysosome size and distribution are clearly altered in tumours (Le Borgne et al., 1998; Steffan et al., 2010), and increased lysosomal migration to the periphery of tumour cells is an important step in metastasis that is related to the lysosomes' normal function in cell migration and wound healing (Fehrenbacher and Jäättelä, 2005). Artificial induction of oncogenic transformation of human breast epithelial cell lines results in altered lysosome structure and function, implying that the effects on lysosomes observed in cancer might be a direct consequence of metabolic transformation (Sloane et al., 1994). Artificial induction also activates the p53-independent, cathepsin-mediated lysosomal cell death pathway, which results in hyper-sensitization to tumour necrosis factor and anti-cancer drugs (Fehrenbacher et al., 2008, 2004), suggesting that altered lysosome biogenesis could be exploited in treating cancer (Fehrenbacher and Jäättelä, 2005). Furthermore, genetic knockdown of LAMPs sensitizes artificially transformed cells to anti-cancer drugs (Fehrenbacher et al., 2008), and inhibiting trafficking of LAMPs to lysosomes activates the cathepsin-mediated lysosomal cell death pathway. Hence, the role we have found for the AP-3 complex in supporting growth of Hap1 myeloid leukemia cells under conditions of acid stress could be related to its role in lysosome biogenesis and trafficking of LAMPs, processes that might already be partially compromised in these cells.

### PAN complex in cancer

The PAN complex (or Pan2–Pan3 complex) is a highly conserved poly(A)-binding protein-dependent poly(A) deadenylase complex that catalyses the initial phase of deadenylation of mRNAs prior to the CCR4-NOT complex-catalysed second phase (Yamashita et al., 2005). Pan2 contains a DEDD family exoRNase domain and is the catalytic subunit, whereas Pan3 confers RNA binding by the complex (Jonas et al., 2014; Schäfer et al., 2014; Wolf et al., 2014). Deadenylation plays an important role in post-transcriptional regulation of mRNA and hence gene expression, by controlling the stability and/or translatability of the mRNA, which is dictated by the length of the poly(A) tail. The PAN complex has also been found to be a component of P-bodies, where it is important for P-body formation through its deadenylase activity (Zheng et al., 2008). Interestingly, a role has been uncovered for the PAN complex in P-bodies, where it promotes translation of the HIF1 protein through stability of *HIF1* mRNA, and expression of HIF1-dependent target genes in response to hypoxic stress (Bett et al., 2013). This is somewhat in contradiction to earlier ideas that P-bodies were sites of mRNA degradation, although it is now becoming increasingly clear that P-bodies and related stress granules play roles in mRNA storage and stress signalling (Anderson et al., 2015). P-bodies and stress granules also have well-established links to cancer, where their composition and abundance are altered in tumour cells, and in response to chemotherapeutic agents (Anderson et al., 2015). The direct involvement of the PAN complex in HIF1 expression provides an enticing possible explanation for our results showing that the PAN complex is required for Hap1 cell growth, given the important role for HIF1 in metabolic transformation and acid stress resistance of cancer cells; and provides a strong rationale supporting that the PAN complex is an important potential target in cancer treatment. Our results also suggest that response to intracellular acid stress might be a previously unrecognized role for P-bodies as part of the cellular stress response.

### Growth phase and acid stress resistance

It was interesting that the acid stress effects in the AP-3 and PAN complex mutants were observed at higher confluence, but not when the Hap1 cells were at lower density and growing exponentially. This is in contrast to the non-tumorigenic HEK293 cells, in which acid stress reduced exponential growth rate. As neither Hap1 nor HEK293 cells showed increased apoptosis under acid stress, this suggests that the decrease in growth rate likely resulted from a slowed cell cycle. Musgrove et al. (1987) have previously found for the PMC-22 human melanoma cell line that cells experiencing intracellular acid stress have a slowed cell cycle and are enriched in quiescent G1-arrested cells. If the HEK293 cells were less capable of counteracting the external acid at media pH 6.8 and 6.4 (perhaps because of fewer acid-extruding mechanisms) and their resulting pH_i_ was lowered as a result, this could explain the observed decrease in exponential growth rate. As the Hap1 cells have likely been selected to be resistant to external acid (during blast formation in the bone marrow) and likely have acid extrusion mechanisms already in place allowing them to maintain optimal pH_i_, this could explain their resistance to acid stress during exponential phase growth. In support of this, Musgrove et al*.* also found that the pH_i_ of PMC-22 cells is maintained during exponential phase growth even though media pH drops as low as pH 6.5 (Musgrove et al., 1987). Curiously, they also found that upon entry into stationary phase the pH_i_ of these same cells plummets. Assuming this drop in pH_i_ also occurs in Hap1 cells approaching stationary phase, our results would suggest that loss of the AP-3 and PAN complexes sensitizes confluent and/or stationary phase cells to this intracellular acid stress, preventing further cell growth.

### Acid stress resistance genes and regulation of intracellular pH

A reasonable explanation for why a gene might be identified to have a role in acid stress resistance would be that the gene participates in regulation of pH_i_. Hence, its deletion would likely result in decreased pH_i_ and increased acid stress because of a defect in pumping acid out of the cell, especially under conditions of extracellular acidification. With the exception of V-ATPase, which has a known direct role in the control of pH_i_ by pumping protons from the cytoplasm into the vacuole, and an indirect role via trafficking of Pma1 to the cell surface (Martínez-Muñoz and Kane, 2008), none of the other genes identified had such known roles. A genome-wide screen of the viable yeast deletion collection for regulators of cytoplasmic pH under both normal and acid-stressing conditions identified 177 genes, and we cross-referenced our list of acid stress resistance genes with these results (Orij et al., 2012). Of the 129 genes we identified, only 12 were in common. There were five genes that when deleted resulted in decreased pH_i_ (*AEP3*, *ATP7*, *HOP2*, *MSS116*, *RRG9*), and none of these were components of the six identified acid stress resistance complexes. Only one gene, *HOP2*, when deleted resulted in decreased pH_i_ in yeast grown under acid stressing conditions on pH 3 medium. *HOP2* encodes a meiosis-specific protein that localizes to chromosomes and prevents synapsis between non-homologous chromosomes, ensuring synapsis between homologs (Hollingsworth et al., 1990). How *HOP2* plays a role in control of cytoplasmic pH is unclear; however, together with our results, a physiological role for *HOP2* in pH regulation seems likely and explains its identification as an acid stress resistance gene. The remaining four genes likely confer acid stress resistance by mechanisms other than control of pH_i_, as their pH_i_ was normal under acid stressing conditions (Orij et al., 2012).

Curiously, seven genes identified in our screen (*COR1*, *CYC3*, *MRPL13*, *MRPL32*, *PET112*, *QCR7*, *SGF73*) were found to have higher pH_i_ than wild type when deleted, when grown on pH 5 medium (Orij et al., 2012). Two of these (*COR1*, *QCR8*) are part of the respiratory chain complex III and one (*PET112*) is part of the glutamyl-tRNA(Gln) amidotransferase complex (Fig. 5). However, none of the corresponding deletion mutants had decreased pH_i_ when grown on pH 3 medium (*SGF73* was not tested). Thus, roles for these genes in pH_i_ control could not explain their role in acid stress resistance as their deletion in fact protects against accumulation of intracellular acid on pH 3 medium by normalizing pH_i_ (or alkalinizing in the case of *MRPL32* and *PET112*). Thus, the majority of genes identified in our screen confer acid stress resistance by mechanisms other than control of pH_i_.

### Mitochondria and acid stress resistance

Our screen identified two complexes with central functions in cellular respiration, mitochondrial respiratory chain complexes III and IV (Fig. 5), as well as factors involved in their assembly and in assembly of the proton transporting ATPase complex (Fig. 3). Additionally, we identified factors responsible for processing of mitochondrial RNA, which are required for translation of two additional mitochondrial-encoded components of the respiratory chain; COB, encoding cytochrome B (a subunit of complex III), and Cox1, a subunit of complex IV. We also identified genes required for initiation of mitochondrial translation and genes encoding subunits of the mitochondrial ribosome (Fig. 3), further suggesting impaired synthesis of mitochondrial respiratory chain complexes as the cause of sensitivity to acid stress in these mutants.

Altered mitochondrial function is well-documented in cancer, and although Warburg's original hypothesis remains largely true, increasing evidence supports that mitochondrial function in tumour cells is often present and required for tumour cell growth and survival (Wallace, 2012). This indicates that in addition to targeting aerobic glycolysis, mitochondrial functions in ATP generation, ROS production and biosynthesis of metabolic precursors are all potential therapeutic targets (Weinberg and Chandel, 2015). Indeed, a drug approved for treating diabetes, metformin, inhibits respiratory chain complex I preferentially in tumour cells and is currently in clinical trials for cancer (Pollak, 2014). A lipid-soluble version, phenformin, is also being tested against tumours that rely on oxidative phosphorylation for growth (Appleyard et al., 2012), as is VLX600, another class of respiratory chain complex inhibitors (Zhang et al., 2014). Our results support that targeting respiratory chain complexes will be a generally useful therapeutic approach, not only for cancer cells capable of performing oxidative phosphorylation, but also for those having the glycolytic phenotype and diminished or absent oxidative phosphorylation, experiencing intracellular acid stress. They also provide an explanation for why respiratory chain complex inhibitors have been found to preferentially affect cancer cells over normal cells (Pollak, 2014), given that cancer cells almost universally experience intracellular acid stress, whereas normal cells relying on oxidative phosphorylation for ATP and ROS generation do not.

How might respiratory chain complexes confer acid stress resistance? Yeast treated with the respiratory chain complex III inhibitor antimycin A showed no change in pH_i_ when grown on a fermentable carbon source (glucose), but when grown on a non-fermentable carbon source (glycerol or ethanol) forcing respiration, showed a large drop in cytoplasmic pH (pH_i_<6) (Orij et al., 2009). Orij et al. (2012) also found in their screen for genes that regulate cytoplasmic pH that disruption of mitochondrial respiratory complexes (complex III included) produces no cytosolic acidification when grown under fermentation conditions at physiological or acid stressing pH, however, under growth conditions forcing respiration results in dramatic intracellular acidification (pH_i_<6) similar to antimycin A treatment. Because our screens were performed on fermentable medium, the role we identified for respiratory chain complexes in acid stress resistance must therefore be separate from their roles in cellular respiration and pH regulation. Interestingly, respiratory chain complex III has been found to play a role in induction of autophagy (Ma et al., 2011). In solid tumours, autophagy is induced in regions that are metabolically stressed, enabling recycling of nutrients, and is required for the survival of these tumour cells (Jin and White, 2008). Hence, a role in autophagy for respiratory chain complexes is a possible explanation for how these complexes promote cell growth under conditions of intracellular metabolic acid stress.

Fascinatingly, a wide variety of mutations in mitochondrial DNA encoding various subunits of all five complexes of the respiratory chain have been found in tumours (Wallace, 2012). For example, the mutation T6777C in the cytochrome c oxidase subunit 1, CO1, has been linked to ovarian cancer (Permuth-Wey et al., 2011). As expected these mutations reduce oxidative phosphorylation and ATP generation; however, the TCA cycle often remains intact, producing critical biosynthetic precursors for cancer cell growth (Weinberg and Chandel, 2015). Our findings that loss of respiratory chain complex function causes yeast cells to be sensitive to intracellular acid stress evokes the hypothesis that tumour cells, as a result of these adaptive mutations in mitochondrial-encoded respiratory chain complexes, might be genetically sensitized to metabolic acid stress. Hence, targeting additional acid stress resistance complexes such as AP-3 or PAN in these tumours might be preferentially deleterious over normal tissue. For tumours in which oxidative phosphorylation is not impaired, combination drug therapy approaches using respiratory chain inhibitors such as metformin with drugs that target additional acid stress resistance complexes could lead to selective killing of these tumours as well.

## MATERIALS AND METHODS

### Yeast genetic screening

A query strain for synthetic genetic array analysis (SGA) that results in a hypomorph of the *PMA1* gene was created by disrupting the *YGL007W* hypothetical open reading frame in the Y7092 background with a cassette conferring resistance to ClonNAT. This strain, called *pma1-007*, was mated to the deletion mutant array (DMA) of ∼4800 haploid yeast strains using standard SGA techniques (Tong and Boone, 2006; Young and Loewen, 2013). Following germination of haploids, control arrays containing only the single DMA gene deletions and experimental arrays containing both the DMA deletion and *Δygl007w* were generated. Copies of each array were then subjected to two rounds of plating on to media buffered at pH 3, 4, 5 and 7, each in triplicate. Media was buffered by adding 50 mM sodium phosphate, 50 mM sodium succinate and adjusted to the final indicated pH with sodium hydroxide. Images of plates were captured using a flatbed scanner and analysed using Balony software to quantify the growth of colonies (Young and Loewen, 2013). Colony sizes were normalized by dividing by the median colony size on each plate and correcting for effects resulting from the position of a colony on the plate. To identify putative acid stress resistance genes, a list was compiled of deletion strains that showed a fitness defect in the context of the *ygl007w* deletion at pH 4.0. Using Balony, the ratio of normalized growth of the double mutants was calculated relative to the growth of the corresponding DMA single deletion strains. In order for a strain to be classified as acid-sensitive this ratio needed to be below an arbitrary cut-off value determined from the standard deviation within the pH 4 screen, which in this case was determined as 0.76. This cut-off had to be met in each of the three replicates, with a *P*-value of <0.05.

### Gene ontology

The ClueGO plugin for Cytoscape was used to determine enrichment of gene ontology terms for the pH 4 acid stress screen. The parameters used were: right-sided hypergeometric test for enrichment with Bonferoni step down; minimum GO level=4, maximum GO level=10; GO grouping=yes; GO fusion=no; Kappa score threshold=0.4; initial group size=2; sharing group %=50.0; group by Kappa statistic=yes; group overview term=smallest *P* value.

### Cell culturing conditions

Hap1 control, AP3D1 (772-12) and PAN2 (454-6) knockout cells were obtained from Horizon Genomics. The AP3D1 knockout clone contains a 2 bp insertion in exon 2 (guide RNA: CATAGCGGTGAAGGCGAACG) whereas the PAN2 knockout contains an 8 bp deletion in exon 2 (guide RNA: TATGTGCACTGATTCCTGGA). Both knockout cell lines were confirmed by Sanger sequencing. Hap1 cells were maintained in Iscove's modified Dulbecco's media (IMDM) media (12440053, Invitrogen) supplemented with 10% FBS. HEK293A cells were maintained in DMEM/F12 medium (Sigma) supplemented with 10% FBS.

### IncuCyte growth assays

Hap1 cells were seeded at 10,000 cells/well density in 96-well plates with IMDM media supplemented with 10% FBS. After 2 h media was replaced with IMDM with 10% FBS at pH 7.2, 6.8 or 6.4. The plates were placed in the IncuCyte Zoom Imager (Essen Bioscience) for live cell imaging. Cells were imaged (4× magnification) every 2 h for at least 72 h using the phase contrast channel. HEK293 cells were cultured similarly except media was DMEM/F12+10% FBS, made up at pH 7.2, 6.8, and 6.4, and cells were imaged for longer because of their slower growth rate (at least 120 h). Cell growth was determined by measuring the percent confluence over time. To determine doubling times for growth at pH 7.2, nonlinear regression analysis was performed using GraphPad Prism software using percentage confluence values from the initial time points up until ∼50% confluence as this time period corresponded to an exponential increase in confluence for each strain. A minimum of six replicate wells were used per time point for each strain and condition. Student's *t*-test was performed to determine significance values.

### MTT assays

Cells were plated in 96-well plates at 10,000 cells/well. The number of viable cells was quantified using a 3-(4,5-dimethylthiazol-2-yl)-2,5-diphenyltetrazolium bromide (MTT; M2128, Sigma) assay at 0, 24, 48, and 72 h. Specifically, MTT was added to each well at 1 mg/ml in regular growth media for 2 h. Viable cells reduce the MTT to form a purple precipitate that is then solubilized by the addition of dimethyl sulfoxide (DMSO). A spectrophotometer plate reader was used to measure optical density at 560 nm, thereby giving an indication of viable cell number.

### Viability assays

Viability was assessed by propidium iodide (PI) exclusion. Cells were plated in 96-well plates and incubated for 24, 48, or 72 h with media at pH 7.2, 6.8, or 6.4. Media containing propidium iodide (Sigma) and Hoechst 33342 (Sigma) was added directly to wells at indicated time points, to final concentrations of 0.25 µg/ml and 0.5 µg/ml, respectively. Plates were incubated with Hoechst and PI at 37°C for 30 min, and then scanned using the Cellomics Arrayscan VTI automated fluorescence imager. The cells were imaged for both Hoechst and PI. The ‘target activation’ algorithm was applied to obtain a nuclear mask in the Hoechst channel (to identify all cells) and a corresponding mask was applied to the PI channel. Cellomics software (Thermo Fisher) was used to calculate percentage of cells that were positive for PI and this was used to calculate percent viability based on PI exclusion. For verification of PI uptake, several wells of cells were incubated with 0.05% Triton X-100 at the time of Hoechst and PI addition. These wells showed 95-100% uptake of PI. As a biological positive control, cells were treated for 24 h with 1 µM staurosporine (Sigma), a known inducer of apoptosis.

### Western blots

Cells were lysed using RIPA buffer (50 mM Tris pH 7.5, 150 mM NaCl, 1% Nonidet P-40, 0.5% sodium deoxycholate) containing protease inhibitors (Roche cOmplete ULTRA tablets) before separation by SDS-PAGE. Anti-delta AP3 rabbit polyclonal antibody (1:1000) was a kind gift from Dr Andrew Peden (The University of Sheffield, Sheffield, UK). Anti-PAN2 polyclonal antibody was purchased from MBL Life Science (RN104PW; 1:1000). Anti-beta actin (Sigma, A4700; 1:1000) was used as a loading control.
